# Insights into the maternal-fetal interface from spatial multi-omics

**DOI:** 10.3389/fcell.2026.1911385

**Published:** 2026-07-29

**Authors:** Xiaowei Wei, Qian Li, Haoyi Jia, Chuanmei Qin, Sifan Zou, Yaxuan Hou, Qihan Guo, Xing Li, Yuan Zhang, Jinfang Wang, Yi Lin

**Affiliations:** 1 Reproductive Medicine Center, Shanghai Sixth People’s Hospital Affiliated to Shanghai Jiao Tong University School of Medicine, Shanghai, China; 2 Department of Pathology, Montefiore Medical Center/Albert Einstein College of Medicine, Bronx, NY, United States; 3 Department of Pathology, Northwell Health, New Hyde Park, NY, United States; 4 Shanghai Jiao Tong University School of Medicine, Shanghai, China; 5 General Hospital of Ningxia Medical University, Yinchuan, China

**Keywords:** maternal–fetal interface, placenta, placenta accreta spectrum, preeclampsia, spatial multi-omics

## Abstract

Recent advances in spatial transcriptomics, spatial proteomics, spatial metabolomics, and multimodal integration strategies have substantially transformed current understanding of the maternal–fetal interface. Rather than functioning as a uniform tissue, the placenta is increasingly recognized as a highly organized multicellular ecosystem in which trophoblast, immune, vascular, and metabolic compartments establish spatially coordinated microenvironments that regulate trophoblast differentiation, immune adaptation, vascular remodeling, and placental homeostasis. Emerging evidence further suggests that placental disorders are driven not simply by trophoblast-intrinsic abnormalities but by dysregulation of localized microenvironmental niches within placental tissues. In particular, preeclampsia (PE) and placenta accreta spectrum (PAS) appear to represent opposite spatial ecosystem states characterized by invasion-restrictive versus invasion-permissive microenvironments, respectively. In this review, we summarize recent spatial multiomic studies focused on the maternal–fetal interface and discuss how spatially resolved approaches are reshaping current understanding of placental disease through a spatial microenvironmental framework.

## 1 Introduction

The maternal–fetal interface is a spatially organized system composed of fetal-derived placental villi and maternal decidual tissues. Syncytiotrophoblasts mediate exchange at the outer villous surface, while cytotrophoblasts serve as progenitor cells and extravillous trophoblasts invade the decidua to remodel spiral arteries. The maternal decidua contains stromal, immune, and vascular cells that collectively regulate trophoblast invasion and immune tolerance (Vento-Tormo et al., 2018; Greenbaum et al., 2023). Successful placentation requires tightly regulated trophoblast differentiation and invasion, immune tolerance, spiral artery remodeling, extracellular matrix organization, and metabolic adaptation within specialized placental microenvironments (Menkhorst et al., 2016; Pijnenborg et al., 2006; Kareus et al., 2026). Disruption of these regulatory processes contributes to major pregnancy disorders, including preeclampsia (PE) and placenta accreta spectrum (PAS), which remain leading causes of maternal and fetal morbidity worldwide (Phipps et al., 2019; Jauniaux et al., 2025).

Traditional bulk transcriptomic and metabolic approaches have provided important insights into placental biology; however, these methods average molecular signals across heterogeneous tissues and therefore obscure cell-type–specific programs, regional signaling networks, and localized pathological alterations (Tekola-Ayele et al., 2022). Although single-cell RNA sequencing (scRNA-seq) has substantially improved characterization of placental cellular diversity and differentiation trajectories, dissociation-based approaches inevitably disrupt tissue architecture and spatial context, limiting understanding of how localized cellular interactions regulate placental development and disease (Xenakis et al., 2026). Recent advances in spatially resolved multi-omics technologies have fundamentally transformed investigation of the maternal–fetal interface by enabling high-resolution molecular profiling while preserving tissue organization (Ounadjela et al., 2024). Emerging evidence further suggests that placental function is dynamically regulated through specialized microenvironmental niches rather than through isolated cell-type–intrinsic programs alone (Arutyunyan et al., 2023; Wei et al., 2025; Bartels et al., 2024a).

In this review, we summarize recent advances in spatial multi-omics technologies and discuss how these approaches are reshaping current understanding of placental biology through a spatial microenvironmental framework. We focus on the spatial organization of the maternal–fetal interface, including trophoblast, immune, stromal, vascular, and metabolic niches, and further discuss how dysregulation of these spatial niches contributes to pregnancy disorders such as PE and PAS. Finally, we highlight emerging directions in placental spatial biology, including multimodal integration, spatial metabolomics, artificial intelligence-assisted analysis, and three-dimensional tissue reconstruction.

A systematic literature search was performed using PubMed, Web of Science, and Google Scholar to identify relevant studies on the maternal–fetal interface and spatial multi-omics in pregnancy-related disorders, particularly preeclampsia and placenta accreta spectrum. The following keywords were used in various combinations with Boolean operators (AND/OR): “maternal–fetal interface”, “syncytiotrophoblast”, “extravillous trophoblast”, “cytotrophoblast”, “decidua”, “placenta”, “spatial transcriptomics”, “spatial proteomics”, “spatial metabolomics”, “spatial multiomics”, “single-cell RNA sequencing”, “preeclampsia”, and “placenta accreta spectrum”. Studies were included if they were original research articles published in peer-reviewed English-language journals and provided mechanistic or spatial insights into placental biology or pregnancy-related disorders. Review articles were included selectively when they provided conceptual or methodological relevance. Non-English publications and studies lacking primary data or mechanistic relevance were excluded. The literature search primarily focused on studies published within the past 5 years, with particular emphasis on recent advances in spatial multi-omics technologies at the maternal–fetal interface.

## 2 Spatial multi-omics approaches for reconstructing the maternal–fetal interface

**FIGURE 1:**
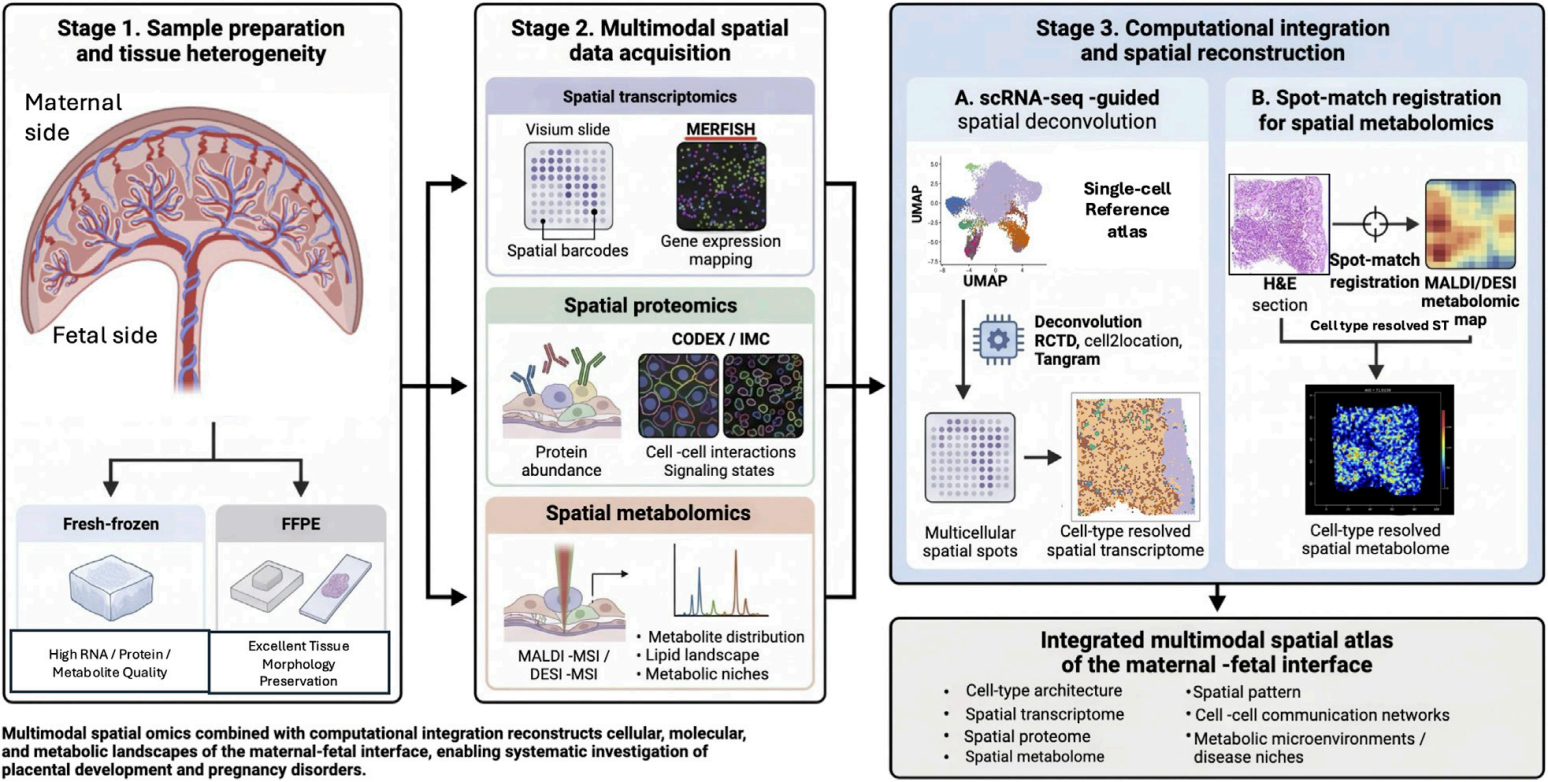
Multimodal Spatial Omics and Computational Integration Workflow at the Maternal–Fetal Interface. Schematic overview of spatial multi-omics technologies and computational integration strategies for reconstructing the maternal–fetal interface. Following tissue collection, placental specimens can be processed as either fresh-frozen or formalin-fixed paraffin-embedded (FFPE) samples, depending on the downstream application. Spatial transcriptomics, spatial proteomics, and spatial metabolomics provide complementary molecular information regarding cellular identity, protein expression, signaling activity, and metabolic states while preserving tissue architecture. Computational integration approaches, including single-cell RNA sequencing (scRNA-seq)-guided spatial deconvolution and spot-match registration between spatial transcriptomic and metabolomic datasets, enable cell type-resolved reconstruction of transcriptomic and metabolic landscapes. Integration of these multimodal datasets generates a comprehensive spatial atlas of the maternal–fetal interface, facilitating the investigation of cellular organization, intercellular communication networks, metabolic microenvironments, and pathological niche formation during placental development and pregnancy disorders.

To dissect the cellular architecture and molecular organization of the maternal–fetal interface, researchers increasingly employ complementary spatial multi-omics technologies, including spatial transcriptomics, spatial proteomics, and spatial metabolomics, each providing unique insights into the tissue microenvironment. Because individual modalities capture distinct biological layers, multimodal integration strategies are increasingly used to associate cellular identity, regulatory programs, signaling activity, and metabolic states within spatial tissue context (Figure 1).

### 2.1 Single-cell RNA sequencing and spatial transcriptomics

Recent advances in spatial transcriptomics (ST) have substantially improved investigation of placental spatial architecture and regional tissue organization at the maternal–fetal interface. ST combines genome-wide transcriptomic profiling with spatial localization, enabling investigation of gene expression directly within intact tissue sections. Current ST platforms can generally be divided into capture-based and imaging-based approaches (Williams et al., 2022). Capture-based technologies, including 10× Genomics Visium, Slide-seq, Stereo-seq, and High-Definition Spatial Transcriptomics (HDST), use spatially barcoded arrays to assign transcriptomic information to defined tissue coordinates and support large-scale profiling across tissue sections. In contrast, imaging-based methods such as MERFISH and seqFISH directly visualize transcripts through iterative fluorescence imaging and achieve near single-cell or subcellular spatial resolution (Liao et al., 2021; Asp et al., 2020).

Integration of scRNA-seq with spatial transcriptomics has become a central strategy for reconstructing tissue cellular architecture and resolving spatial heterogeneity in complex tissues (Huang et al., 2024; Shi et al., 2025). Because capture-based spatial transcriptomic platforms such as 10× Visium generate transcriptomic profiles from multicellular spots rather than true single cells, computational integration with scRNA-seq reference datasets is required to infer the spatial distribution of individual cell types (Tian et al., 2023). In a typical workflow, scRNA-seq is first used to identify transcriptionally distinct cell populations and generate reference cell-type signatures, after which deconvolution algorithms map these signatures back onto spatial transcriptomic data to estimate the abundance and localization of each cell type across tissue regions. Representative computational frameworks include RCTD [(Cable et al., 2022)], Stereoscope (Andersson et al., 2020), Tangram (Biancalani et al., 2021), SPOTlight (Elosua-Bayes et al., 2021), and Cell2Location (Kleshchevnikov et al., 2022), each employing distinct probabilistic or machine-learning strategies for spatial mapping and cell-type inference. Such computational frameworks are particularly valuable in placental tissues, where trophoblasts, stromal cells, endothelial cells, and immune populations are spatially intermingled within highly heterogeneous maternal–fetal microenvironments.

Beyond spatial transcriptomics alone, recent technical updates successfully integrate *in situ* transcriptomic profiling with spatial epigenomics. For instance, combining single-nucleus multi-omics with submicrometre-resolution sequencing or multiplexed imaging allows researchers to map chromatin accessibility alongside gene expression at single-cell resolution. Such multimodal frameworks are instrumental for dissecting cell-type-specific gene-regulatory networks within complex tissue microenvironments, as discussed in detail below (Wang C. et al., 2026; Wang H. et al., 2026).

Overall, spatial transcriptomics provides a powerful approach for dissecting the cellular heterogeneity and spatial architecture of the placenta, offering new insights into maternal–fetal interactions in both health and disease.

### 2.2 Spatial proteomics

Spatial proteomics has emerged as an essential tool because protein activity and subsequent biological functions are heavily dictated by subcellular and tissue-scale localization rather than static abundance alone—nuances that are typically obscured by the averaged signals of conventional bulk approaches (Wang C. et al., 2026; Wang H. et al., 2026; Bernardini et al., 2026). Given that proteins can exhibit divergent or even opposing functional roles depending on their compartmentalization (e.g., nuclear vs. cytoplasmic), preserving this spatial architecture is critical for accurately mapping signaling pathways, metabolic states, and intercellular communication networks *in situ* (Bernardini et al., 2026; Method of the Year 2024: spatial proteomics, 2024; Wu M. et al., 2024).

While spatial transcriptomics has transformed our understanding of cellular organization at the maternal–fetal interface, many critical biological processes, including immune recognition, cell adhesion, and trophoblast invasion, are ultimately mediated at the protein level. Spatial proteomic approaches therefore provide complementary insights by resolving cell–cell interactions, signaling states, and tissue architecture *in situ* (Bartels et al., 2024a). Multiplexed spatial proteomic platforms, including CODEX, MIBI, and imaging mass cytometry (IMC), enable simultaneous visualization of dozens of immune and stromal markers within intact tissue architecture. These approaches have revealed highly organized immune niches at the maternal–fetal interface, where uterine NK cells, macrophages, and trophoblasts establish spatially restricted interactions that contribute to immune tolerance and vascular remodeling (Bartels et al., 2024a). Compared with transcriptomic approaches, spatial proteomics can capture signaling activation states and post-transcriptional regulation, offering unique advantages for investigating inflammatory signaling, hypoxia responses, and immune checkpoint regulation within placental microenvironments (Bartels et al., 2024a).

### 2.3 Spatial metabolomics

Spatial metabolomics enables direct characterization of localized metabolic activities within intact tissue sections and provides functional biochemical information that cannot be fully inferred from transcriptomic or proteomic analyses alone. Because gene and protein expression do not necessarily correlate with metabolic pathway activity, spatial metabolomics offers unique opportunities to investigate region-specific metabolic states, lipid remodeling, and biochemical microenvironmental organization within complex tissues (Alexandrov, 2023; Hu et al., 2023). Current spatial metabolomic approaches are primarily based on imaging mass spectrometry technologies, including matrix-assisted laser desorption/ionization mass spectrometry imaging (MALDI-MSI) and desorption electrospray ionization mass spectrometry imaging (DESI-MSI), which allow *in situ* detection of metabolites and lipids while preserving tissue architecture (Alexandrov, 2023; Luo et al., 2023).

Recent advances in quantitative spatial metabolomics have further improved interpretation of imaging mass spectrometry datasets through isotope-labelled internal standard normalization and pixel-wise quantitative correction strategies (Wang et al., 2025). Spatial isotope tracing approaches additionally provide opportunities to investigate localized metabolic flux, nutrient utilization dynamics, and pathway activity during trophoblast differentiation and placental remodeling (Li X. et al., 2025).

To align these metabolic landscapes with cellular identities, investigators have recently integrated spatial metabolomics with transcriptomic profiling. Cross-platform registration methods, such as coordinate-transformation-based ‘spot-matching’ strategies, facilitate point-to-point alignment of metabolomic and transcriptomic datasets across adjacent tissue sections (Wei et al., 2025). This cross-modal integration enables the direct correlation of localized biochemical alterations with specific cellular lineages and differentiation trajectories.

### 2.4 Tissue preservation strategies for spatial multi-omics

Tissue preservation strategy is an important consideration in spatial multi-omics studies because RNA integrity, metabolite stability, tissue morphology preservation, and platform compatibility are highly dependent on sample processing methods. Fresh-frozen tissues generally preserve higher RNA quality and native metabolite composition, making them particularly suitable for spatial transcriptomics and spatial metabolomics analyses, especially for studies investigating lipid remodeling and metabolic microenvironments (Bhalla et al., 2023). In contrast, formalin-fixed paraffin-embedded (FFPE) tissues provide superior tissue morphology preservation and broad clinical accessibility but often exhibit RNA fragmentation and partial metabolite degradation during fixation and embedding procedures (Williams et al., 2022).

Selection of tissue preservation methods should therefore be guided by the biological objectives and analytical platforms used in individual studies. Fresh-frozen tissues remain the preferred choice for metabolome-preserving spatial analyses, whereas FFPE-compatible technologies increasingly facilitate large-scale clinical and translational placental research (Wei et al., 2025; Liu et al., 2022). Such sample selection principles are critical for subsequent spatial niche exploration in physiological and pathological placental tissues (Table 1).

**TABLE 1:** Suitability of fresh-frozen and FFPE placenta for three types of spatial multi-omics.

Spatial omics type	Fresh frozen placenta	FFPE placenta
Spatial transcriptomics	Gold standard	Available (poor RNA yield)
Spatial metabolomics	Mandatory choice	Not feasible
Spatial proteomics	Best epitope preservation	Partial only (antigen loss)

## 3 Spatial organization of the maternal–fetal interface

The maternal–fetal interface (MFI) is a dynamic and highly organized structure formed through the interaction of maternal and fetal tissues. It primarily comprises the chorionic villi, anchoring villi, basal plate, decidua basalis, intervillous spaces, and remodeled spiral arteries. These compartments collectively support embryo implantation, placental development, maternal–fetal exchange, immune tolerance, and host defense throughout pregnancy (Huang et al., 2023). The maternal component is represented by the decidua, which serves as the principal site of trophoblast invasion and spiral artery remodeling. This region contains decidual stromal cells, natural killer cells, macrophages, dendritic cells, and T cells that coordinate immune adaptation and regulate trophoblast behavior (Kareus et al., 2026). The fetal component consists mainly of chorionic villi bathed in maternal blood within the intervillous space. Villi are covered by syncytiotrophoblasts (SCT), which mediate nutrient and gas exchange while forming an immunologically protective barrier (Wang C. et al., 2026). Beneath this layer, cytotrophoblasts (CTB), vascular endothelial cells, and Hofbauer cells contribute to placental development and immune homeostasis (Gauster et al., 2022; Schust et al., 2021). EVTs at the anchoring villi and basal plate displayed invasive and extracellular matrix remodeling signatures, consistent with their role in maternal tissue invasion and spiral artery remodeling (Wang C. et al., 2026). Together, these cellular and structural elements establish a specialized microenvironment that is essential for maintaining a successful pregnancy [(Kareus et al., 2026)].

Placental development is further influenced by localized biochemical and biophysical gradients across the maternal–fetal interface. Oxygen availability, nutrient distribution, inflammatory signaling, extracellular matrix composition, and angiogenic factor expression vary substantially across placental regions and dynamically shape trophoblast differentiation, immune adaptation, vascular remodeling, and metabolic activity (Vornic et al., 2024a; Vornic et al., 2024b; Mandalà, 2025). The intervillous space represents a particularly important microenvironment characterized by direct maternal blood exposure, oxygen fluctuations, and metabolic exchange. Collectively, these observations support the concept that placental development and disease are governed by tissue ecosystems rather than uniformly distributed tissue-wide processes. Apart from human samples, spatial omics in mouse and porcine models also facilitate cross-species exploration of maternal-fetal structural development (McCutcheon et al., 2025; Wu Y. et al., 2024).

## 4 Spatial microenvironmental niches at the maternal–fetal interface

### 4.1 Trophoblast niches

Recent spatial multiomic studies reconstructed trophoblast lineage trajectories *in situ* at the maternal-fetal interface (Wei et al., 2025). Wang et al. provide an unprecedented single-cell spatiotemporal atlas of the human MFI across gestation, capturing over 1.1 million cells spanning early to late pregnancies. Using integrated single-nucleus RNA sequencing and single-nucleus ATAC-seq, the authors identified diverse cellular populations, including VCTs, SCTs, and EVTs, as well as stromal, immune, and endothelial cells, with precise maternal versus fetal origin assignments. Pseudotime and trajectory analyses revealed the bifurcation of VCTs into SCT and EVT lineages, highlighting anchoring villi VCTs as EVT progenitors and floating villi VCTs as SCT progenitors (Wang C. et al., 2026). Spatial regulon analyses further revealed that SCT-associated programs are enriched for hormone secretion, nutrient transport, and endoplasmic reticulum stress pathways, whereas EVT-associated programs are linked to epithelial–mesenchymal transition and extracellular matrix organization, indicating that trophoblast lineage specification is spatially coupled to specialized functional niches (Liu et al., 2022).

EVTs arise from VCT progenitors through a tightly controlled differentiation process that involves the acquisition of an invasive phenotype and the progressive loss of epithelial characteristics. Following differentiation within trophoblast cell columns, EVTs exit the cell cycle and migrate away from the anchoring villi into the surrounding decidua. These cells can follow two principal migratory pathways. Interstitial EVTs (iEVTs) infiltrate the decidual stroma and interact extensively with maternal immune cells and extracellular matrix components. In contrast, endovascular EVTs (eEVTs) invade the lumens and walls of spiral arteries, replacing maternal endothelial cells and contributing directly to vascular remodeling (Chang et al., 2018). During the first trimester, EVTs temporarily occlude the distal portions of spiral arteries, thereby limiting maternal blood flow into the intervillous space. This physiological restriction protects the developing placenta and embryo from excessive oxidative stress and hemodynamic forces while allowing nutrients and signaling molecules derived from maternal plasma and uterine gland secretions to reach the conceptus (Li Q. et al., 2025; Weiss et al., 2016).

By integrating spatial transcriptomics with single-cell data, Arutyunyan et al. generated a spatially resolved single-cell atlas spanning placental, decidual, and myometrial compartments during early pregnancy and reconstructed EVT differentiation trajectories *in situ*. They identified EVT-2 as a critical branching state from which distinct EVT subtypes emerge and revealed divergent regulatory programs associated with EVT fate specification, highlighting the activation of TGFβ signaling and suppression of Wnt pathways in iEVTs and enhanced Notch signaling and reduced TGFβ activity in eEVTs (Arutyunyan et al., 2023). Wang et al. performed single-cell transcriptomic profiling of the human full-term maternal–fetal interface and proposed PRDM6 as a potential regulator of eEVT differentiation. Furthermore, reduced PRDM6 expression and impaired epithelial-to-mesenchymal transition–related signaling were associated with defective EVT differentiation and vascular remodeling in preeclampsia (Wang et al., 2022).

Spatial multiomic analyses revealed that trophoblast differentiation is governed by complex gene-regulatory networks involving extensive cis-regulatory element (CRE)–gene interactions. CTBs maintained progenitor identity through TP63-, TFAP2-, TEAD4-, and FOXP1-associated programs, whereas EVT differentiation was characterized by activation of AP-1, EMT-related, and MYCN-centered invasive pathways. In contrast, STB differentiation was associated with enrichment of NFE2, CEBP family members, ATF4, and ESRRG, indicating distinct transcriptional mechanisms governing syncytialization. Notably, chromatin accessibility of several lineage regulators decreased upon terminal differentiation, suggesting dynamic epigenetic remodeling during trophoblast maturation. Functional studies further identified FOXP1 as a key regulator of trophoblast stemness, maintaining progenitor states while suppressing premature syncytial differentiation. Together, these findings provide a comprehensive regulatory framework for trophoblast lineage commitment and maturation (Ounadjela et al., 2024).

Spatial analyses additionally demonstrated that trophoblast differentiation is coordinated through localized signaling interactions within villous and decidual microenvironments. SCTs and VCTs exhibit extensive ligand–receptor interactions within villous regions, highlighting their central role in coordinating trophoblast differentiation and placental homeostasis. Spatial co-localization of PGF–FLT1 and WNT-associated signaling pathways further suggests that trophoblast behavior is regulated through localized developmental signaling hubs rather than uniformly distributed molecular cues. WNT5A and WNT7A were predominantly enriched within SCT-associated regions, whereas receptors including FRZB and LDLR displayed broader distribution across villous compartments, supporting the existence of spatially restricted trophoblast regulatory niches (Liu et al., 2022). The study further identified DLK–NOTCH signaling between fibroblasts and trophoblast populations. DLK expression was enriched in fibroblasts, whereas NOTCH2 and NOTCH3 receptors were broadly expressed in trophoblast populations, suggesting spatially coordinated stromal–trophoblast communication within the villous core. IGF2–IGF2R interactions were also spatially enriched within trophoblast-associated regions. IGF2 expression was particularly abundant in fibroblasts and VCTs, whereas IGF2R was broadly distributed across villous cell populations (Liu et al., 2022).

Collectively, these findings support the concept that trophoblast differentiation occurs within multicellular communities shaped by localized stromal, vascular, immune, and signaling microenvironments at the maternal–fetal interface.

### 4.2 Immune niches

Spatial multiomic profiling revealed that immune regulation at the maternal–fetal interface is highly dependent on spatial organization rather than the abundance of individual immune cell populations alone. Vento-Tormo et al. demonstrated that decidual NK (dNK) cells, macrophages, decidual stromal cells (DSCs), endothelial cells, and invading trophoblasts establish highly localized immune niches characterized by regional cytokine gradients, immune checkpoint signaling, and short-range ligand–receptor communication networks. Distinct immune microenvironments are established around trophoblast invasion routes and decidual compartments, creating localized niches that support fetal tolerance while preserving immune surveillance (Ounadjela et al., 2024). Early gestational tissues are enriched in cytotoxic NK subsets and pro-inflammatory macrophages, whereas later stages show progressive accumulation of tolerogenic macrophages expressing CD163, CD206, TIM-3, galectin-9, and IDO1, reflecting temporal remodeling of immune function. Spatial co-localization of GAL-9+ fibroblasts with CD57^+^ NK cells additionally suggests that stromal compartments actively participate in establishment of immunoregulatory microenvironments (Greenbaum et al., 2023). Recent spatial transcriptomics studies in human decidua further demonstrated that these immunoregulatory niches are not only defined by cell type composition but also by spatially coordinated differentiation and signaling programs. Sha et al. showed that the implantation zone is enriched in dNK1 cells and dM2 macrophages, forming a tightly organized niche with extensive ligand-receptor interactions, including collagen-mediated and IL-15 signaling pathways. They found that dNK2 cells normally differentiate toward dNK1 cells in this niche, a process driven by IL-15 and transcription factor FOSL2. In recurrent pregnancy loss, this niche is disrupted, with loss of dNK1 and dM2 populations, impaired cell colocalization, and expansion of cytotoxic CD8^+^ T cells, highlighting how spatial disorganization of immune networks can compromise maternal-fetal tolerance (Sha et al., 2026). Krop et al. used imaging mass cytometry technology and revealed that myeloid cells constitute a dominant component of the maternal-fetal immune niche throughout gestation. HLA-DR^-^ myeloid subpopulations were the most frequent neighbors of EVTs in first-trimester decidua, highlighting their potential role in supporting trophoblast invasion and maternal-fetal tolerance. Myeloid cell subclusters dynamically interacted with NK cells and T cells over gestation, complementing spatial transcriptomic findings on dNK1/dM2 niches and providing a more complete view of immune-trophoblast crosstalk (Krop et al., 2022).

dNK cells and macrophages directly interact with EVTs via localized ligand–receptor signaling, including PVR–TIGIT, CSF1–CSF1R, and CCR1–CCL5 axes, supporting trophoblast differentiation, migration, and invasion (Greenbaum et al., 2023; Wei et al., 2022). Decidual macrophages and dendritic cells complement dNK functions by maintaining tissue homeostasis, promoting trophoblast differentiation, and producing cytokines such as IL-10 and TGF-β to establish a tolerogenic environment. T cells within the decidua are skewed toward regulatory and memory phenotypes, with CD4^+^ Tregs and CD8^+^ tissue-resident T cells spatially localized near trophoblast invasion sites, further reinforcing immune tolerance (Kareus et al., 2026).

The EVT lineage also demonstrated enrichment of several genes associated with immune modulation and maternal-fetal communication. For example, ATP11A, which contributes to the generation of a “don’t-eat-me” signal on the cell surface, may help EVTs evade maternal immune surveillance. Other genes, including FGFR1, QSOX1, and DIO2, were identified as potential regulators of trophoblast invasiveness, immune interactions, and local thyroid hormone signaling (Ounadjela et al., 2024). Importantly, Greenbaum et al. highlight that immune cells act as active regulators rather than passive components, shaping local trophoblast behavior and microenvironmental homeostasis (Greenbaum et al., 2023).

Emerging evidence additionally suggests that immune regulation at the maternal–fetal interface extends beyond classical immune cell populations. Placental erythroid cells and innate lymphoid cells were shown to express immune checkpoint molecules, antigen-presentation machinery, cytokines, and chemokines, supporting potential roles in immune suppression, immune cell recruitment, antimicrobial defense, and local microenvironmental regulation within placental tissues (Greenbaum et al., 2023; Kareus et al., 2026). Collectively, these findings indicate that maternal–fetal immune tolerance is maintained through highly localized multicellular signaling ecosystems within decidual tissues.

Spatial transcriptomic and proteomic analyses identified substantial heterogeneity among decidual stromal populations, including subsets associated with extracellular matrix remodeling, angiogenesis, endocrine signaling, and immune modulation, supporting active regulatory functions of stromal compartments within placental tissues (Wang C. et al., 2026). In addition, Wang et al. discovered a previously unrecognized decidual stromal population expressing CNR1, the cannabinoid receptor 1. Functional experiments demonstrated that this DSC subtype suppresses EVT invasion through endocannabinoid signaling, suggesting that maternal stromal cells actively regulate trophoblast behavior rather than simply serving as a structural scaffold. This finding reveals a novel mechanism controlling the balance between adequate trophoblast invasion and excessive placental infiltration (Wang C. et al., 2026).

### 4.3 Spiral artery remodeling niches

Spatial community analysis defined six recurrent microenvironments, including decidual niches, maternal arterial niches, fetal villous vascular niches, floating villi, and the basal plate, each with distinct cell-type compositions and functional specialization (Wang C. et al., 2026). Spiral artery remodeling (SAR) represents one of the most specialized vascular niches at the maternal–fetal interface. A detailed morphometric analysis of early human pregnancy demonstrated that this process occurs in a highly dynamic and spatially heterogeneous manner. eEVT progressively infiltrate the arterial lumen and form trophoblast plugs, while iEVT invade the surrounding decidua and contribute to perivascular remodeling. Concurrently, vascular smooth muscle cells and extracellular matrix components are gradually lost, resulting in luminal expansion and conversion of high-resistance spiral arteries into low-resistance uteroplacental vessels (Zhang, 2020). Importantly, endothelial replacement proceeds progressively, with endothelial integrity partially preserved during early remodeling stages before eventual displacement by trophoblasts (Zhang, 2020).

Wang et al. provides direct molecular evidence for EVT-mediated spiral artery remodeling by demonstrating that maternal arterial endothelial cells undergo a stepwise transition rather than being passively displaced. Spatial transcriptomic and protein-level analyses identified a continuum of endothelial states (caEC→R0→R1→R2), characterized by progressive loss of endothelial junctions, suppression of arterial identity genes, detachment from the vessel wall, and activation of apoptotic programs. Importantly, the proximity of EVTs correlated with endothelial state progression, suggesting that trophoblast invasion actively drives endothelial destabilization and clearance. These findings establish a mechanistic framework in which EVTs transform high-resistance spiral arteries into dilated, low-resistance vessels capable of sustaining adequate placental perfusion (Wang C. et al., 2026).

Greenbaum et al. established a detailed five-stage SAR classification system based on arterial morphology, smooth muscle integrity, endothelial continuity, and EVT infiltration. They showed that SAR progression only partially correlated with gestational age, indicating that vascular remodeling is strongly influenced by local tissue microenvironments rather than occurring uniformly throughout the uterus (Greenbaum et al., 2023). NicheNet analyses identified JAG1–NOTCH, LEP–LEPR, and CD24-associated signaling pathways between EVTs and arterial cells, implicating localized trophoblast–vascular interactions in endothelial replacement, smooth muscle loss, vascular dilation, and arterial remodeling. Notably, genes involved in Notch signaling progressively decreased during SAR, whereas pathways associated with smooth muscle remodeling and angiogenesis exhibited biphasic temporal dynamics. These results illustrated that spiral artery remodeling is not a single event but rather a coordinated multistage biological process involving temporally regulated molecular programs (Greenbaum et al., 2023).

Emerging evidence demonstrated that dNK cells and decidual macrophages actively participate in vascular remodeling. These immune populations produce angiogenic factors, cytokines, and matrix-remodeling enzymes, including VEGF, angiopoietins, and matrix metalloproteinases, which contribute to vascular smooth muscle cell disruption, extracellular matrix degradation, and endothelial modification (Albrecht and Pepe, 2020). Experimental studies further suggest that immune cells may initiate early stages of vascular transformation before extensive trophoblast invasion occurs (Ashkar et al., 2000; Care et al., 2018). In addition, trophoblast-derived signals can recruit and activate immune cells around remodeling vessels, highlighting the importance of trophoblast–immune–vascular crosstalk during placentation (Wei et al., 2022).

### 4.4 Spatial metabolic microenvironments

Emerging evidence suggests that spatial communication within tissues extends beyond canonical ligand–receptor signaling and additionally involves metabolite-mediated and hormone-mediated information flow. Integration of spatial multiomics may therefore provide new opportunities to investigate metabolic communication networks at the maternal–fetal interface. Advanced multi-modal mass spectrometry imaging has revealed that placental villi are highly compartmentalized metabolic microenvironments. Using an integrated workflow combining MALDI-based lipidomics, on-tissue chemical derivatization (OTCD)-enhanced metabolomic imaging, and microscale proteomics, Armingol et al. mapped the STB and villous core compartments at micrometer resolution. The STB is enriched for steroidogenesis, fatty acid transport, and β-oxidation, whereas the villous core preferentially supports ketone body utilization and extracellular matrix-associated metabolism, highlighting the existence of compartment-specific metabolic niches within placental villi (Veličković et al., 2025). Proteomic analyses confirmed compartment-specific enzyme distributions, including steroidogenic enzymes (CYP11A1, HSD3B1), ceramide biosynthesis enzymes (SPTLC1, KDSR), and ketolytic enzymes (BDH2, ACAT1). Integration of metabolite and protein localization allowed reconstruction of active metabolic pathways *in situ*, demonstrating that STB and villous core maintain distinct lipid and energy metabolism programs (Veličković et al., 2025).

Spatially resolved multi-omics has begun to unravel the intricate lipid and metabolic reprogramming characterizing pregnancy complications. For instance, Chen et al. integrated single-cell sequencing with spatial transcriptomics and metabolomics to reveal profound immune-metabolic dysregulation in preeclamptic placentas, highlighting cell-type-specific alterations in lipid transport and cholesterol homeostasis (e.g., APOE, APOC1, and ABCA1) within the trophoblast and Hofbauer cell niches (Chen Y. et al., 2026). In addition, our group utilized a novel ‘spot-match’ strategy to couple Visium spatial transcriptomics with DESI-based spatial metabolomics, generating a cell-type-resolved multi-omic landscape of the late-onset preeclamptic maternal–fetal interface (Wei et al., 2025). This approach uncovered lineage-specific glycerophospholipid and sphingolipid dynamics along the VCT-to-SCT and VCT-to-EVT differentiation pathways, where dysregulation of key enzymes like LPL and DGKZ directly tied to the localized accumulation of diacylglycerols and depletion of phosphatidylcholine derivatives (Wei et al., 2025). Complementing spatial multi-omics studies of human placental metabolic niches, Fu et al. constructed a spatiotemporal transcriptomic atlas of the mouse placenta, revealing that glycogen trophoblast cells (GCs) dynamically migrate from the junctional zone to the maternal decidua during late gestation. Using functional experiments in Ano6 knockout mice, the authors showed that impaired GC glycogen catabolism led to excessive glycogen accumulation, reduced levels of key metabolites (glucose) in placenta and fetal liver, and perinatal lethality, while maternal glucose supplementation partially rescued fetal survival. These findings demonstrate that spatially resolved transcriptomics combined with functional validation can uncover how cell type-specific metabolic activities directly support fetal viability and highlight the essential role of localized metabolic niches at the maternal-fetal interface (Fu et al., 2026).

**FIGURE 2:**
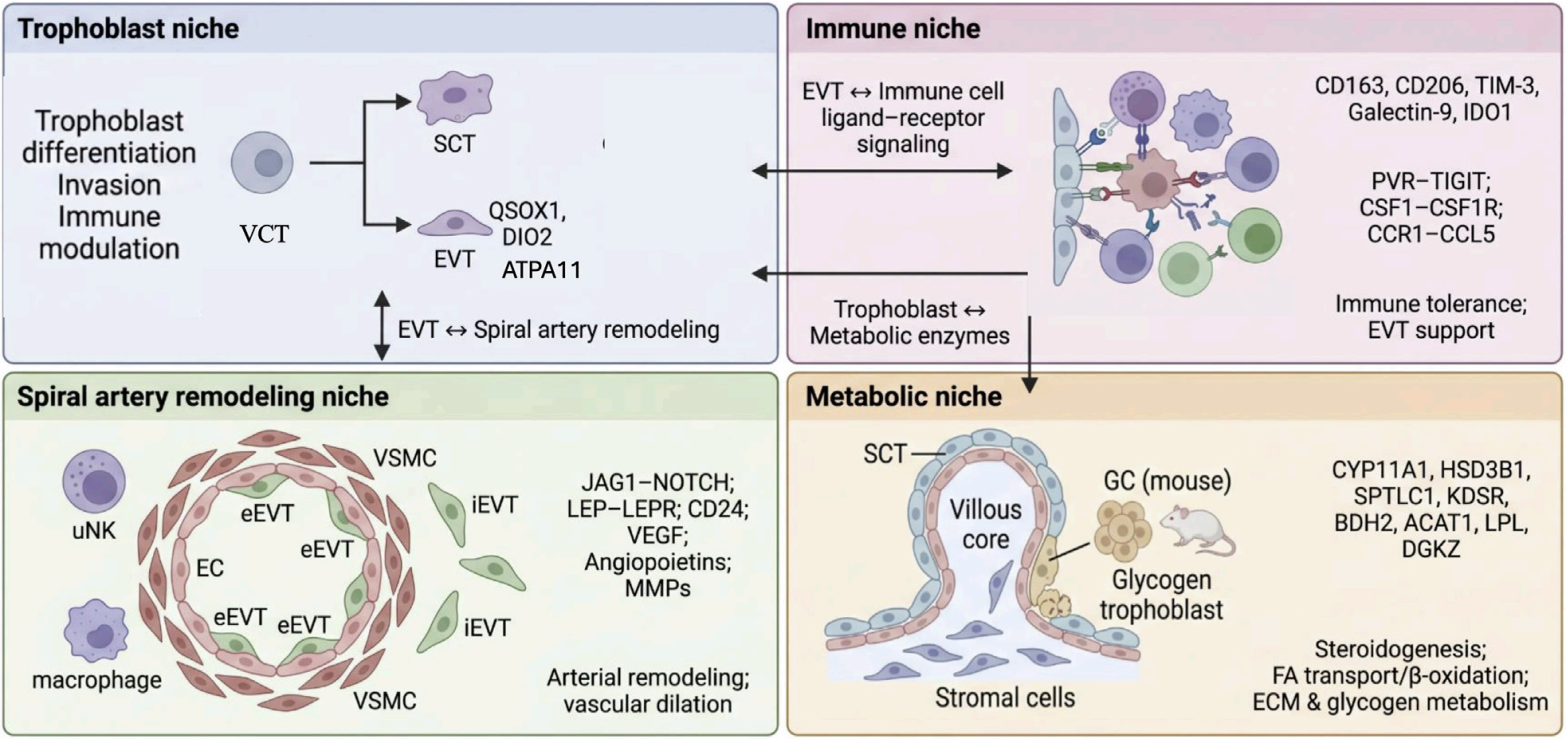
Spatial microenvironmental niches and key markers at the maternal-fetal interface. An integrative schematic showing four specialized functional domains (Top Left) Trophoblast niche: Trophoblast differentiation lineages (VCT, SCT, EVT) and associated markers governing invasion and differentiation (Top Right) Immune niche: Maternal immune cells, tolerance-associated markers (e.g., CD163, TIM-3), and active ligand-receptor signaling pairs supporting EVTs (Bottom Left) Spiral artery remodeling niche: Cellular components (eEVT, iEVT, VSMC, EC) and signaling cascades (e.g., JAG1-NOTCH, VEGF, MMPs) driving vascular transformation and dilation (Bottom Right) Metabolic niche: Placental villous architecture and key metabolic enzymes regulating steroidogenesis, lipid transport, and glycogen metabolism. Arrows denote cross-niche signaling and interactions.

Although direct spatial metabolomics remains technically challenging, computational approaches have emerged as complementary tools for reconstructing tissue-scale metabolic landscapes. Using the scCellFie framework, Armingol et al. demonstrated that metabolic activities exhibit pronounced cell type-specific and spatially organized patterns, with distinct metabolic programs linked to tissue remodeling, cellular differentiation, and metabolite-mediated intercellular communication (Armingol et al., 2025). In the endometrium, scCellFie identified dynamic metabolic remodeling across the menstrual cycle, including coordinated regulation of glycolysis, nucleotide salvage, kynurenine metabolism, and phenylalanine metabolism in epithelial and stromal compartments. Furthermore, spatial analyses uncovered metabolite-mediated intercellular communication networks, particularly involving the kynurenine–AHR signaling axis, which was associated with tissue remodeling and cellular homeostasis. These findings highlight the potential of transcriptome-derived metabolic inference to complement spatial metabolomics and facilitate the identification of localized metabolic microenvironments within complex tissues (Armingol et al., 2025). Collectively, these studies demonstrate that metabolic activities are spatially organized across trophoblast, stromal, vascular, and immune compartments, and that disruption of these localized metabolic niches may contribute to placental dysfunction and pregnancy complications (Figure 2).

## 5 Opposite spatial microenvironmental disorders

**FIGURE 3:**
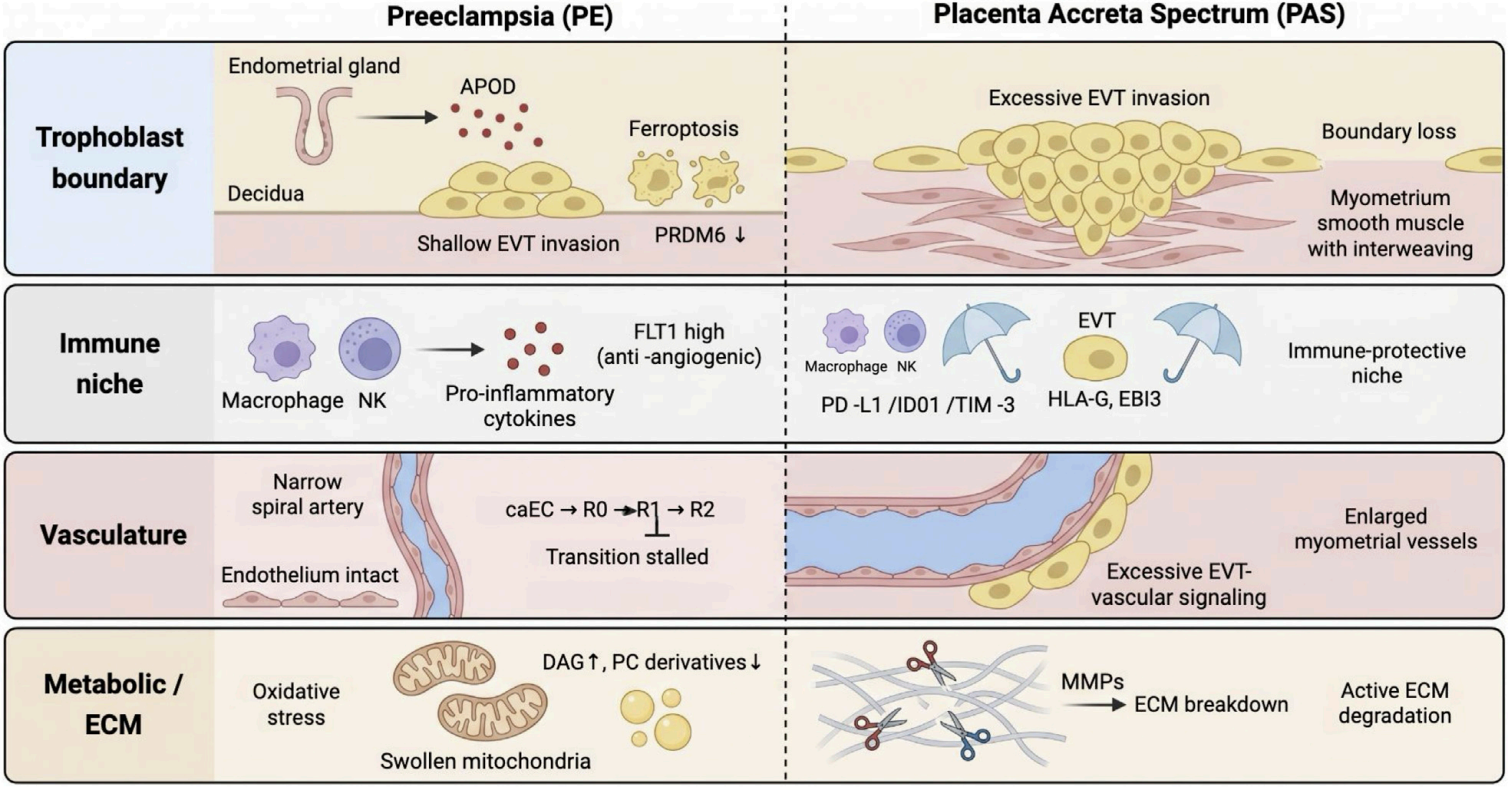
The Yin–Yang Microenvironmental Ecosystem: Invasion-Restrictive Preeclampsia versus Invasion-Permissive Placenta Accreta Spectrum. Conceptual model illustrating preeclampsia (PE) and placenta accreta spectrum (PAS) as opposite spatial ecosystem states at the maternal–fetal interface. The maternal–fetal microenvironment is organized into four interconnected niches: trophoblast, immune, vascular, and metabolic/extracellular matrix (ECM) compartments. On the PE side (left), trophoblast invasion is restricted, characterized by reduced PRDM6-associated EVT differentiation, shallow trophoblast invasion, and APOD-mediated ferroptosis induced by endometrial gland-derived signals. Immune niches exhibit inflammatory activation with macrophages, NK cells, pro-inflammatory cytokines, and elevated FLT1 signaling. Vascular remodeling is impaired, resulting in narrow high-resistance spiral arteries, endothelial dysfunction, and stalled endothelial state transitions during spiral artery remodeling. Metabolically, oxidative stress, mitochondrial dysfunction, diacylglycerol accumulation, and depletion of phosphatidylcholine derivatives contribute to an invasion-restrictive microenvironment. In contrast, the PAS side (right) is characterized by excessive EVT invasion and loss of the decidual boundary, allowing direct interaction between trophoblasts and myometrial smooth muscle cells. An immunosuppressive invasion niche is established through trophoblast expression of HLA-G and EBI3 and increased PD-L1 (CD274), IDO1, and TIM-3 (HAVCR2) signaling in surrounding stromal and immune cells. Excessive EVT–vascular communication promotes abnormal enlargement and remodeling of myometrial vessels. Concurrently, active ECM degradation and remodeling mediated by matrix metalloproteinases (MMPs) create a permissive microenvironment that facilitates pathological placental invasion. Together, these opposing niche ecosystems highlight the concept that PE and PAS represent two extremes of trophoblast invasion biology, driven by coordinated dysregulation of trophoblast, immune, vascular, and metabolic microenvironments rather than isolated cellular defects.

Proper placentation depends on tightly coordinated interactions between trophoblasts and the maternal decidual microenvironment. Disruption of these spatially organized cellular and molecular networks can lead to a spectrum of pregnancy disorders (Vornic et al., 2025). Notably, preeclampsia (PE) and placenta accreta spectrum (PAS) can be viewed as opposite ends of the trophoblast invasion continuum. While PE is associated with shallow placental invasion, PAS is characterized by excessive placental attachment and deep myometrial invasion (Bartels et al., 2024b). Recent advances in spatial multi-omics have enabled *in situ* characterization of these local regulatory networks, revealing disease-specific alterations in stromal, vascular, immune, and extracellular matrix niches (Kim et al., 2026; Tang et al., 2024) (Figure 3).

### 5.1 Preeclampsia

PE is a complex pregnancy-specific syndrome characterized by the development of hypertension after mid-gestation, frequently accompanied by proteinuria and/or evidence of maternal organ dysfunction (Fox et al., 2019). Affecting approximately 2%–8% of pregnancies worldwide, PE remains one of the leading causes of maternal and perinatal morbidity and mortality (Jeyabalan, 2013). PE can present as early- or late-onset forms, with distinct origins and clinical outcomes. Early-onset preeclampsia (EOPE, before 34 weeks) is primarily linked to defective placental development, while late-onset preeclampsia (LOPE, 34 weeks or later) is more strongly associated with metabolic factors (Kariori et al., 2025). Differentiating these subtypes is important for understanding disease mechanisms and for guiding timely diagnosis, risk assessment, and individualized management.

Solt et al. generated a comprehensive single-cell and single-nucleus transcriptomic atlas comprising approximately 90,000 placental cells across 46 cell types and states from EOPE, LOPE, and gestational age-matched controls. Their analyses revealed profound dysregulation of trophoblast, stromal, endothelial, and immune compartments in EOPE, including FLT1/PGF angiogenic imbalance, inflammatory activation, stromal stress responses, and vascular dysfunction. In contrast, LOPE exhibited comparatively limited placental transcriptional alterations, with most changes restricted to specific cell populations and pathways. These findings support the concept that distinct preeclampsia subtypes involve different molecular mechanisms and microenvironmental remodeling patterns (Solt et al., 2025). In addition, Campbell et al. constructed a large placental single-cell RNA sequencing reference atlas containing over 40,000 cells from healthy term placentas and used it to deconvolute bulk placental transcriptomic datasets from preeclampsia. Using CIBERSORTx, the authors demonstrated that preeclamptic placentas exhibit substantial alterations in cellular composition, including increased extravillous trophoblasts and reduced Hofbauer cells and mesenchymal stromal cells (Campbell et al., 2023; Hartmann et al., 2023). Similarly, spatial deconvolution by Chen et al. demonstrated a striking accumulation of EVTs within the cytotrophoblast shell of PE placentas. While the corresponding region in normal placentas contained a balanced composition of endovascular EVTs, interstitial EVTs, and trophoblast giant cells, the PE trophoblast shell was predominantly occupied by EVTs. Histological examination and immunofluorescence staining for HLA-G further confirmed extensive EVT deposition within this niche (Chen M. et al., 2026).

Spatial multi-omics further reveals that preeclampsia is not a uniform disorder but a spectrum of spatially and functionally distinct placental pathologies. Chen et al. performed an integrative multi-omics study to investigate subtype-specific immune-metabolic remodeling in EOPE and LOPE. The study analyzed placental tissues from EOPE, LOPE, and gestational age-matched controls, together with serum samples collected during early pregnancy from an independent cohort of 199 women. They found that EOPE is characterized by disrupted oxygen transport, enhanced hypoxia signaling, and compensatory angiogenic responses, whereas LOPE exhibits stronger inflammatory signaling and extracellular matrix remodeling. Importantly, metabolic changes were not uniform across disease subtypes. EOPE exhibited upregulation of lipid transport pathways, consistent with a compensatory response to hypoxia, whereas LOPE showed downregulation of these pathways, indicative of metabolic exhaustion. This subtype-specific divergence highlights a dynamic shift from adaptive metabolic reprogramming to decompensated mitochondrial dysfunction during disease progression (Chen Y. et al., 2026). This study also identified 14 major placental cell types and revealed substantial alterations in cellular composition and differentiation states in preeclampsia. EOPE showed increased macrophages and extravillous trophoblasts, alongside reduced oxygen-transporting Hofbauer subtypes, whereas LOPE largely preserved cellular composition but displayed pronounced inflammatory transcriptional programs (Chen Y. et al., 2026). EOPE is a disorder not only of placental development but also of maternal microenvironmental dysfunction. Excessive secretion of apolipoprotein D (APOD) from endometrial glands disrupts early trophoblast function and spiral artery remodeling, ultimately leading to placental insufficiency.

Notably, elevated APOD levels were detectable in first-trimester maternal serum prior to disease onset, suggesting that maternal-derived metabolic factors may serve as early predictive biomarkers and therapeutic targets (Dong et al., 2025). Collectively, these studies demonstrate that preeclampsia is characterized by coordinated remodeling of trophoblast, immune, vascular, and metabolic microenvironments rather than isolated defects in individual cell populations. The integration of single-cell, spatial transcriptomic, and spatial metabolomic approaches is therefore providing a more comprehensive framework for understanding PE heterogeneity and identifying novel targets for diagnosis and intervention. These circulating lipid and APOD-related biomarkers may provide new opportunities for first-trimester non-invasive screening of EOPE.

### 5.2 Placenta accreta spectrum

PAS refers to a group of disorders in which placental tissue abnormally attaches to or invades the myometrium, leading to difficult placental separation at delivery. The incidence of PAS has increased markedly with rising cesarean section rates and is associated with substantial maternal morbidity due to severe hemorrhage (Jauniaux et al., 2025). The molecular and cellular mechanisms underlying PAS have yet to be fully elucidated.

Bartels et al. employed integrated immunohistochemistry, spatial transcriptomics, and spatial proteomics to characterize the maternal–fetal interface in PAS. The authors demonstrated that abnormal placental invasion is associated with a spatially distinct microenvironment marked by altered immune cell distribution, enhanced extracellular matrix remodeling, and increased trophoblast invasiveness. Spatial analyses revealed activation of AP-1 and NF-κB signaling, upregulation of ECM-degradation pathways, and enrichment of immunosuppressive factors, including PD-L1/PD-L2, BCL-2, and CD14^+^ macrophages. Furthermore, ligand–receptor interaction analysis identified fibronectin–ITGβ1 signaling as a key driver of EVT invasion and tissue remodeling. These findings highlighted the molecular pathways, particularly fibronectin–ITGB1 signaling and immune checkpoint activation, that facilitate pathological trophoblast invasion in PAS(13).

By jointly resolving trophoblast populations, stromal compartments, immune cells, and their spatial organization, Ji et al. moved beyond conventional trophoblast-centered models and demonstrated that PAS is associated with the formation of a spatially localized immunosuppressive invasion niche (Ji et al., 2024). Compared with non-invasive placental regions, PAS invasion sites are characterized by elevated EVT proportion and diminished syncytiotrophoblast populations. At invasive fronts, trophoblasts directly contact myometrial smooth muscle due to the loss of physiological decidual barrier (Ji et al., 2024). The study provided novel evidence that PAS invasion sites exhibit a uniquely immunosuppressive microenvironment. Increased expression of HLA-G and EBI3 in trophoblasts, together with elevated CD274 (PD-L1), IDO1, and HAVCR2 expression in smooth muscle and immune cell compartments, suggested coordinated establishment of localized immune tolerance and immune escape at the invasion front (Ji et al., 2024). Afshar et al. also revealed that the most prominent transcriptional alterations occurred in endothelial and stromal populations rather than trophoblasts alone, with dysregulation of extracellular matrix, angiogenesis, and growth factor–related genes including COL3A1, EGFL6, HGF, DLK1, DCN, SPARC, and SPP1. Spatial transcriptomics further demonstrated substantial intraplacental heterogeneity between adherent and nonadherent regions and identified extensive ECM remodeling and abnormal decidual–vascular niche signaling in PAS. These findings support a conceptual shift from the traditional “excessive trophoblast invasion” model toward a “loss of boundary limits” model driven by dysfunctional stromal and endothelial microenvironments (Afshar et al., 2024).

## 6 Discussion

Spatial multi-omics has transformed our understanding of the maternal–fetal interface by revealing highly organized trophoblast, immune, vascular, and metabolic niches. Evidence from PE and PAS demonstrates that abnormal placentation is driven not only by trophoblast-intrinsic defects but also by dysregulation of surrounding microenvironments (Muñoz-Blat et al., 2025; Mangla et al., 2026). These findings support a spatial ecosystem framework for interpreting placental development and disease [(Ounadjela et al., 2024; Wang H. et al., 2026; Soncin, 2025; Moore et al., 2022)].

### 6.1 Limitations and challenges of spatial multi-omics

Despite the rapid expansion of spatial transcriptomic and spatial multiomic technologies, several major technical and biological challenges continue to limit the derivation of mechanistic insight from spatial profiling datasets. One of the primary limitations involves trade-offs among spatial resolution, transcriptome coverage, sensitivity, and tissue-scale profiling. Imaging-based platforms achieve near single-cell or subcellular resolution but are often restricted by limited transcript panels and imaging complexity, whereas sequencing-based approaches provide broader transcriptome coverage at the cost of multicellular capture spots and reduced spatial precision (Wu et al., 2026). In addition, current spatial transcriptomic studies frequently capture only limited tissue regions, which may inadequately represent the large-scale spatial heterogeneity of the maternal–fetal interface. Tissue preservation introduces additional logistical hurdles, particularly given the placenta’s high susceptibility to ischemia-associated RNA degradation and rapid post-delivery metabolic shifts. While the historical trade-offs between fresh-frozen (optimal for multi-omics) and FFPE tissues (optimal for morphology) were delineated in earlier sections, the ongoing challenge lies in scaling FFPE-compatible spatial platforms to facilitate high-throughput, retrospective clinical trials without sacrificing transcriptomic sensitivity or metabolite integrity (Garcia-Alonso et al., 2021; Bilous et al., 2026).

Another major challenge involves accurate reconstruction of cellular architecture and intercellular communication within complex tissue microenvironments. Despite substantial advances in deconvolution frameworks, accurate reconstruction of cell boundaries and mixed-cell signals remains challenging (Shi et al., 2025). Furthermore, computational inference of cell–cell communication is inherently limited by the fact that transcript abundance does not necessarily reflect protein secretion, receptor activation, or downstream signaling activity. Although emerging frameworks such as CellPhoneDB v5 increasingly incorporate spatial proximity, transcription factor activity, and multiomic integration, many predicted signaling interactions still require experimental validation (Troulé et al., 2025). Importantly, recent studies further suggest that tissue communication extends beyond canonical ligand–receptor interactions and additionally involves metabolite-mediated, lipid-mediated, and hormone-mediated signaling networks, emphasizing the need for integrative spatial metabolomic and proteomic analyses (Gong and Weinberg, 2025; Li Z. et al., 2025).

Current spatial transcriptomic approaches are also fundamentally constrained by their predominantly two-dimensional representation of highly complex three-dimensional tissues. This limitation is particularly important for placental biology, as trophoblast invasion, villous branching, spiral artery remodeling, and maternal–fetal immune interactions occur within dynamic three-dimensional architectures that continuously evolve throughout gestation. Future advances in the field will likely focus on multimodal spatial integration, three-dimensional tissue reconstruction, dynamic lineage tracing, and longitudinal spatiotemporal profiling. In parallel, emerging computational frameworks increasingly conceptualize spatial transcriptomic data as tissue niches or multicellular ecosystems rather than isolated cell populations, thereby enabling more biologically interpretable reconstruction of tissue organization and microenvironmental regulation. Although spatial multiomic technologies have substantially improved characterization of placental cellular ecosystems, many inferred signaling interactions remain descriptive and require functional validation. Integration of spatial omics with trophoblast organoid systems, perturbation experiments, and gene-editing approaches may provide powerful platforms for mechanistic investigation of trophoblast differentiation, immune regulation, and placental disease pathogenesis (McCutcheon et al., 2025; Vento-Tormo, 2023).

### 6.2 Translational implications and future perspectives

Spatial multi-omics provides a spatially resolved framework for linking local microenvironmental alterations at the maternal–fetal interface to systemic circulating biomarkers. By characterizing cell type–and niche-specific molecular programs within placental tissues, including trophoblast, immune, and metabolic compartments, spatial analyses may help infer the potential tissue and cellular sources contributing to circulating signals. These spatially defined molecular features can be partially reflected in placenta-derived cell-free DNA (cfDNA), cell-free RNA (cfRNA), and circulating metabolites in maternal peripheral blood, which serve as non-invasive indicators of trophoblast turnover, immune activation, and metabolic remodeling. Although the direct causal mapping between spatial tissue signatures and circulating biomarkers remains to be fully established, integration of spatial multi-omics with clinical and computational approaches may facilitate improved interpretation of peripheral biomarkers and support the development of predictive models for pregnancy-related disorders such as preeclampsia and fetal growth restriction.

Looking forward, integration of spatial transcriptomics with spatial proteomics, metabolomics, epigenomics, and artificial intelligence-based analytical frameworks may substantially improve mechanistic understanding of placental biology and disease pathogenesis. In particular, combining spatial multiomics with functional validation strategies, including trophoblast organoids, perturbation experiments, lineage tracing, and gene-editing approaches, may help distinguish causal regulatory mechanisms from descriptive spatial associations. These advances may ultimately facilitate identification of spatial biomarkers for pregnancy complications, improve pathological classification of placental disorders, and support development of personalized therapeutic strategies targeting compartment-specific microenvironments at the maternal–fetal interface. In the future, spatially specific placental biomarkers are expected to be translated into pathological auxiliary diagnosis and individualized targeted therapy for PE and PAS patients.

## Glossary

MFI: Maternal–fetal interface
PE: Preeclampsia
EOPE: Early-onset preeclampsia
LOPE: Late-onset preeclampsia
PAS: Placenta accreta spectrum
scRNA-seq: Single-cell RNA sequencing
snRNA-seq: Single-nucleus RNA sequencing
snATAC-seq: Single-nucleus Assay for Transposase-Accessible Chromatin using sequencing
ST: Spatial transcriptomics
SCT: Syncytiotrophoblast
CTB: Cytotrophoblast
VCT: Villous cytotrophoblast
EVT: Extravillous trophoblast
iEVT: Interstitial extravillous trophoblast
eEVT: Endovascular extravillous trophoblast
DSC: Decidual stromal cell
dNK: Decidual natural killer cell
NK: Natural killer cell
Treg: Regulatory T cell
VSMC: Vascular smooth muscle cell
EC: Endothelial cell
SAR: Spiral artery remodeling
ECM: Extracellular matrix
PGF: Placental growth factor
FLT1: Fms-like tyrosine kinase 1 (VEGFR1)
VEGF: Vascular endothelial growth factor
APOD: Apolipoprotein D
IL: Interleukin
TGF-β: Transforming growth factor beta
WNT: Wingless-related integration site signaling pathway
NOTCH: Notch signaling pathway
JAG1: Jagged canonical Notch ligand one
MMP: Matrix metalloproteinase
HLA-G: Human leukocyte antigen G
IDO1: Indoleamine 2,3-dioxygenase one
PD-L1: Programmed death-ligand 1 (CD274)
TIM-3: T cell immunoglobulin and mucin domain-3 (HAVCR2)
MALDI-MSI: Matrix-assisted laser desorption/ionization mass spectrometry imaging
DESI-MSI: Desorption electrospray ionization mass spectrometry imaging
CODEX: CO-Detection by indEXing
IMC: Imaging mass cytometry
MIBI: Multiplexed ion beam imaging
PGF–FLT1 axis: Placental growth factor–VEGFR1 signaling axis
